# Efficacy and Safety of Anticoagulants in Patients With Idiopathic Pulmonary Fibrosis: A Meta‐Analysis

**DOI:** 10.1155/carj/4545181

**Published:** 2025-12-14

**Authors:** Shuo Yang, Di Xie, Jinyun Wang, Zhuomin Yang, Xiaoming Xue

**Affiliations:** ^1^ Department of Respiration, Institute of Shanxi Traditional Chinese Medicine, Taiyuan, 030012, China; ^2^ Department of Traditional Chinese Medicine, Shanxi University of Traditional Chinese Medicine, Taiyuan, 030619, China, sxtcm.com; ^3^ Department of Respiration, Shanxi Province Hospital of Traditional Chinese Medicine, Taiyuan, 030012, China

**Keywords:** 6MWT, adverse events, anticoagulants, D-dimer, DLCO, FVC, IPF, meta-analysis

## Abstract

**Background:**

There is a known association between coagulation abnormalities and idiopathic pulmonary fibrosis (IPF), but it remains unclear whether anticoagulant treatment can extend the lifespan of IPF patients. This systematic review and meta‐analysis aim to evaluate the efficacy and safety of anticoagulants in patients with IPF.

**Methods:**

A comprehensive search was conducted in PubMed, Embase, Cochrane, and Web of Science from their inception until June 30, 2024, with an English language restriction. We selected clinical studies that met the inclusion criteria after reviewing titles, abstracts, and full texts.

**Results:**

Three randomized controlled trials and five prospective studies, including 2185 patients, were analyzed. Compared to standard treatment, anticoagulants did not significantly affect overall mortality in IPF patients. However, excluding studies with warfarin monotherapy, other anticoagulants extended patient survival (OR = 0.56, 95% CI [0.33, 0.95], *p* = 0.03). Anticoagulants were associated with significantly increased adverse events (OR = 2.48, 95% CI [1.16, 5.32], *p* = 0.68) but improved lung function (forced vital capacity, FVC), increased lung capacity, and reduced plasma D‐dimer levels. No significant differences were observed in the 6‐min walk test (6MWT) or diffusing capacity of the lungs for carbon monoxide (DLCO).

**Conclusion:**

The study suggests that anticoagulants may extend the lifespan of IPF patients and improve lung function. However, they also increase the risk of adverse events such as bleeding. However, there is an increased risk of adverse events, such as bleeding. This may be due to the distinct mechanisms of action, risk profiles, and clinical outcomes of each drug, although the exact causes remain unclear. Further meta‐analyses based on individual patient data are needed to confirm these findings.

## 1. Introduction

Idiopathic pulmonary fibrosis (IPF) is a prevalent interstitial lung disease characterized by poor prognosis and limited life expectancy, with an incidence that continues to rise annually [1]. Clinically, it manifests as progressive dyspnea and varying degrees of dry cough, ultimately leading to respiratory failure [2, 3]. Although significant progress has been made in understanding the pathogenesis of IPF, effective therapeutic strategies remain elusive. To address this clinical challenge, antifibrotic agents are currently the mainstay of treatment. Among them, pirfenidone and nintedanib are commonly prescribed and can partially slow the decline in lung function and alleviate dyspnea. However, studies have shown that their efficacy varies among individuals and that both drugs may cause adverse effects such as gastrointestinal discomfort and skin rash. Some patients require dosage adjustments due to poor tolerance, and neither drug can completely halt disease progression. Despite advances in understanding the pathogenesis of IPF, effective treatments remain elusive [4–6].

In addition to disease progression and therapeutic limitations, abnormalities in coagulation function among patients with IPF have recently attracted growing research interest. Several studies have demonstrated that IPF patients exhibit a hypercoagulable state and face a higher risk of developing venous thromboembolism compared with the general population. A case‐control study further revealed that IPF patients have a greater prevalence of a prothrombotic state [7] than healthy individuals [8, 9]. However, there is still debate regarding the use of prophylactic anticoagulation in IPF patients. Earlier studies, such as those by Kubo et al., linked elevated plasma D‐dimer levels to mortality in patients with acute exacerbation of IPF. The combination of anticoagulants like heparin or warfarin with corticosteroids was shown to reduce mortality in these patients [10]. Nevertheless, the role of prophylactic anticoagulant therapy in this patient population remains controversial. However, a subsequent double‐blind, placebo‐controlled trial by Noth et al. found no benefit of warfarin in IPF treatment, with a potential increase in mortality risk [11]. These two perspectives are contradictory and may be linked to a relationship between thrombosis and fibrosis. Anticoagulants may have a potential effect in reducing thrombosis, improving pulmonary microcirculation, and slowing the progression of fibrosis. However, anticoagulants also increase the risk of bleeding, particularly in patients with underlying thrombotic conditions, which is a significant concern for IPF patients. Therefore, the role of anticoagulants in IPF treatment remains uncertain and requires further investigation.

In animal models of IPF induced by bleomycin (BLM) in rats or mice, elevated plasma D‐dimer levels were observed. Studies confirmed that anticoagulants reduced lung interstitial collagen deposition in these models, suggesting a potential antifibrotic effect [12, 13]. In clinical trials, elevated plasma D‐dimer levels have been associated with increased mortality in patients with acute IPF exacerbations. This suggests that thrombosis may play a role in the progression of IPF, particularly in areas affected by alveolitis or fibrosis [14]. While pulmonary embolism is recognized as a cause of death in patients with IPF, the role of the coagulation system in IPF has received limited attention [9]. To date, no specific treatment for IPF exists, and current pharmacological therapies offer limited efficacy. There is a gap in our understanding of anticoagulant use in these patients. Therefore, we conducted a meta‐analysis to evaluate the effectiveness and safety of anticoagulants in IPF patients.

This meta‐analysis examined eligible trials to assess whether anticoagulant use extends the lifespan of IPF patients and to evaluate the efficacy and safety of anticoagulant therapy.

## 2. Methods

### 2.1. Registration and Protocol

This meta‐analysis followed the PRISMA guidelines for systematic reviews and meta‐analyses [15]. The study was registered with PROSPERO (Registration No.: CRD42024581923).

### 2.2. Search Strategy

We conducted a systematic literature search in electronic databases, including PubMed, Embase, Cochrane, and Web of Science, for studies published up to May 20, 2025. The search was limited to English‐language studies. The strategy was based on keywords and MeSH terms such as “idiopathic pulmonary fibrosis” and “anticoagulants”. The detailed search strategy is provided in Appendix 1. Reference lists of relevant articles were also reviewed to identify additional studies.

### 2.3. Study Selection

The included studies met the following criteria: (1) patients had a clinical diagnosis of IPF [16]; (2) they were controlled trials; (3) the experimental group received anticoagulants of any form, dosage, or duration in addition to standard treatment, while the control group received standard treatment or placebo; and (4) accurate data were obtained after the trial’s completion.

Exclusion criteria included (1) reviews, meta‐analyses, case reports/letters, and conference abstracts; (2) trials with unavailable or missing data without explanation; and (3) studies with duplicate reporting of the same patient cohort.

### 2.4. Outcomes

The primary outcome was the impact of anticoagulants on all‐cause mortality in IPF patients. Secondary outcomes included the safety of anticoagulants, particularly the incidence of bleeding events, forced vital capacity (FVC), diffusing capacity of the lungs for carbon monoxide (DLCO), six‐minute walk test (6MWT), and plasma D‐dimer levels.

### 2.5. Data Extraction and Quality Assessment

After removing duplicates, two researchers (Z.M.Y. and J.Y.W.) independently screened the titles and abstracts for relevance and extracted the data. Discrepancies were resolved by a senior researcher (D.X.). Demographic and outcome data were extracted, including study design, sample size, mean age, sex, all‐cause mortality, bleeding events, lung function (FVC, DLCO), plasma D‐dimer levels, and 6MWT results. All‐cause mortality was assessed at specific time points, categorized as short‐term (3 months), mid‐term (1 year), or with unspecified follow‐up duration. Bleeding events reported in the included studies were not classified according to severity. Changes in lung function (FVC and DLCO) were evaluated relative to baseline values. Study quality and risk of bias were assessed using the ROBINS‐I tool for non‐randomized intervention studies [17].

### 2.6. Data Synthesis

Statistical analysis was performed using Review Manager 5.4.3. Odds ratios (OR) were used as the effect measure for categorical data, with 95% confidence intervals (CI). Statistical significance was set at *p* < 0.05. The I^2^ test was used to assess heterogeneity among studies [18]. If I^2^ > 50%, a random‐effects model was used; if fewer studies were included, a fixed‐effects model was chosen. However, when the study samples have different characteristics, such as age, gender, or treatment duration, using a random effects model for continuous variables better captures these differences. When significant heterogeneity exists, a funnel plot can be used to assess the risk of publication bias. If meta‐analysis was not feasible, a descriptive analysis was performed.

## 3. Results

### 3.1. Eligible Studies and Study Characteristics

**Figure 1:**
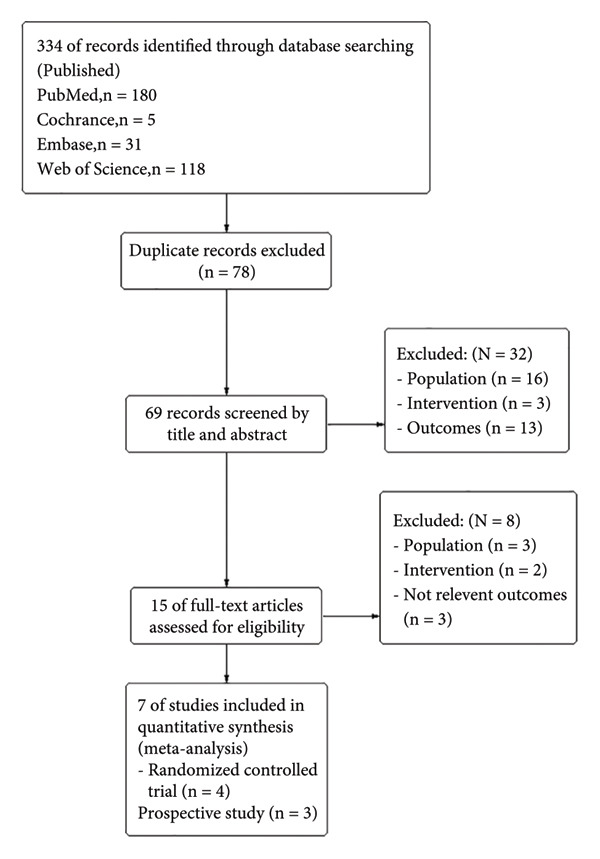
Flow diagram of study selection.

The PRISMA flowchart of the meta‐analysis is shown in Figure 1. Initially, we screened 334 studies. After reviewing titles, abstracts, and full texts, 7 trials [10, 11, 19–23] were included in the final meta‐analysis. These trials involved 2185 participants: 323 patients in the anticoagulant treatment group and 1862 patients in the standard treatment group. The details of the included trials, including the number of participants, sex, mean age, and primary outcomes, are summarized in Table 1.

**Table 1:** Include basic information about the article.

Author	Published time	Country	Design	Age (year)	Sex (male/female)	Patients, n	Outcomes
Mitsuhiro Abe	2015	Japan	Single‐center prospective study	71 ± 9.53	19/3	22	90‐day patient survival rate
Michael Kreuter	2016	Germany	Randomized controlled trials	67.12 ± 7.52	465/159	624	1‐year patient survival rate
Toru Arai	2020	Japan	Prospective study	73.37 ± 9.19	65/20	85	Patient survival rate
Yasuhiro Kondoh	2020	Japan	Randomized, double‐blind placebo‐controlled study	72.04 ± 8.08	67/10	77	90‐day patient survival rate
Christopher S. King	2021	USA	Randomized controlled trials	67.53 ± 10.19	1149/762	1911	Effect of DOACs on mortality risk in patients with IPF
Christopher S. King	2021	USA	Randomized controlled trials	67.55 ± 10.19	1139/772	1911	Effect of warfarin on mortality risk in patients with IPF
Imre Noth	2012	USA	Double‐blind, randomized, placebo‐controlled trial	66.99 ± 7.23	106/39	145	Patient survival rate
Hiroshi Kubo	2005	Japan	Prospective study	69.41 ± 10.11	31/25	56	Effect of anticoagulants on survival in patients with IPF

### 3.2. Quality Assessment

**Figure 2:**
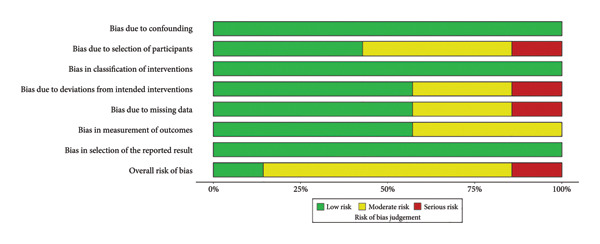
Summary of bias risk.

**Figure 3:**
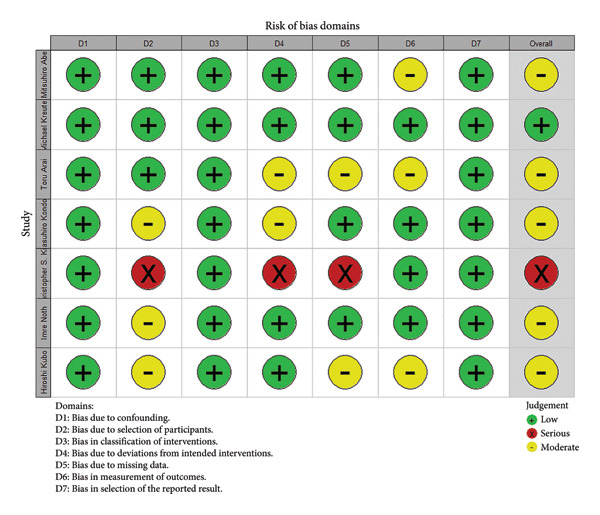
Traffic light figure of bias risk.

Using the ROBINS‐I tool, we identified one study with a low risk of bias, five studies with a moderate risk of bias, and one study with a high risk of bias (Figures 2 and 3).

### 3.3. Primary Outcome: All‐Cause Mortality

**Figure 4:**
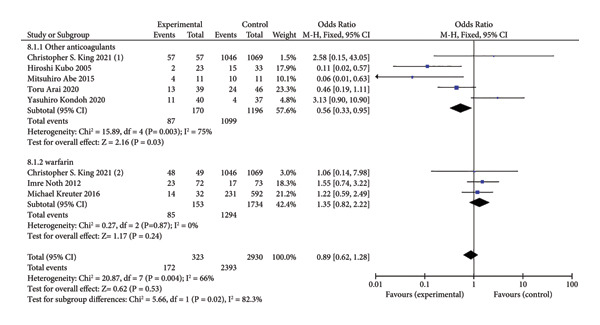
Forest plot of all‐cause mortality.

All‐cause mortality was reported in 8 trials. Of the 170 patients treated with anticoagulants, excluding the warfarin monotherapy group [10, 20, 22], 87 died (51.1%), compared to 1099 deaths among 1196 patients in the control group (91.8%). There was a statistically significant difference between the anticoagulant and control groups (OR = 0.56, 95% CI [0.33, 0.95], I^2^ = 75%, *p* = 0.03; Figure 4). The observed high heterogeneity may be due to differences in anticoagulant type, dosage, or treatment duration, as well as variations in follow‐up time points indicating that anticoagulants effectively reduce mortality in IPF patients compared to standard care or placebo. However, three trials [11, 19, 23] examining the effects of oral warfarin alone found no statistically significant difference in mortality among IPF patients.

### 3.4. Secondary Outcomes

#### 3.4.1. Adverse Effects

**Figure 5:**
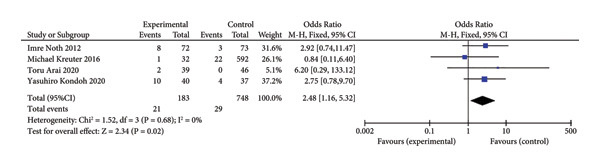
Forest plot of adverse effects.

Events four trials involving 931 participants [11, 20, 22, 23] reported adverse events (bleeding) during anticoagulant therapy. Among the 183 patients in the anticoagulant group, 21 (11.5%) experienced adverse events, while 29 (3.9%) of 748 patients in the control group experienced similar events. A comparison between the two groups showed a significant increase in bleeding risk in the anticoagulant group (OR = 2.48, 95% CI [1.16–5.32]; I^2^ = 0%; Figure 5). This suggests that anticoagulant use in the hypercoagulable state of IPF patients significantly increases the risk of bleeding events.

### 3.5. Lung Function

**Figure 6:**
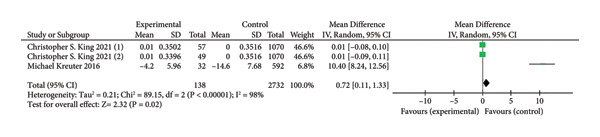
Forest plot of lung function.

Three trials involving 2870 participants [19, 23], reported improvements in lung function based on continuous variable analysis. The anticoagulant group consisted of 138 participants, while the control group included 2732 participants. A comparison between the two groups showed that anticoagulants significantly increased the rate of FVC improvement from pretreatment to post‐treatment stages in IPF patients (MD = −0.72,95% CI [0.11–1.33]; I^2^ = 59%, *p* = 0.02; Figure 6). The high heterogeneity may be attributable to differences in anticoagulant type, dosage, or treatment duration.

### 3.6. 6MWT

**Figure 7:**
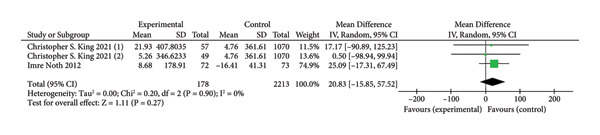
Forest plot of 6WNT.

Three trials involving 2391 participants [11, 19], assessed improvements in the 6MWT based on continuous variable analysis. The anticoagulant group had 178 participants, while the control group had 2213 participants. A comparison revealed that anticoagulants did not significantly improve exercise capacity in patients (MD = 20.83,95% CI [−15.82–57.52]; I^2^ = 0%, *p* = 0.27; Figure 7).

### 3.7. DLCO

**Figure 8:**
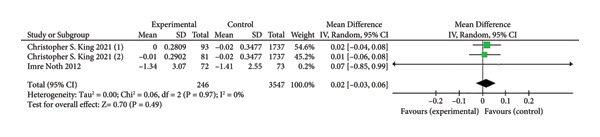
Forest plot of DLCO.

Three trials involving 3793 participants [11, 19] reported on the improvement in DLCO based on continuous variable analysis. The anticoagulant group included 246 participants, while the control group had 3547 participants. A comparison between the two groups showed that anticoagulants did not significantly improve lung function (MD = 0.02, 95% CI [‐0.03–0.06]; I^2^ = 0%, *p* = 0.49; Figure 8).

### 3.8. Publication Bias

**Figure 9:**
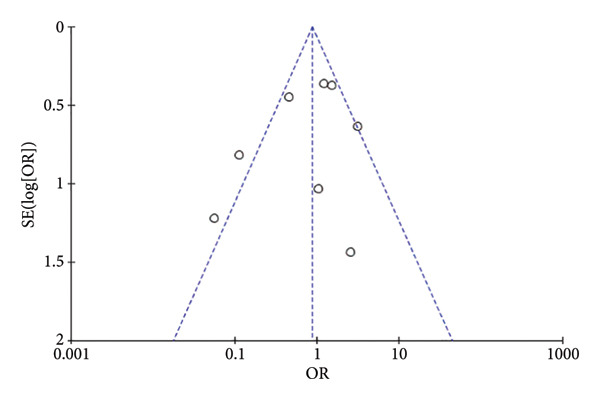
Publication bias.

Eight studies that included all‐cause mortality were selected, and a funnel plot was used to assess the risk of publication bias. The funnel plot for mortality (Figure 9) was nearly symmetrical, indicating no risk of publication bias.

## 4. Discussion

In this meta‐analysis of eight controlled trials involving patients with IPF, previous studies have reported inconsistent conclusions and substantial heterogeneity regarding all‐cause mortality, with particularly notable controversy surrounding the use of oral warfarin. To clarify the impact of different anticoagulant choices on mortality in IPF patients, we analyzed three separate trials involving oral warfarin. The results showed no statistically significant difference in mortality rates. This contrasts with earlier studies that opposed warfarin treatment for IPF [24–26]. These discrepancies may be due to the limited number of included studies or heterogeneity among IPF patient populations in terms of age distribution, comorbidities, and disease severity. The mean age of participants ranged from 62 years (Kubo et al., 2005) to 68 years (Kreuter et al., 2016), with notable differences in comorbidity profiles. In Kreuter et al., 32% of patients had hypertension and 18% had diabetes, whereas Abe et al. (2015) did not explicitly exclude patients with pulmonary hypertension. Since comorbid hypertension and diabetes are known to impair vascular endothelial integrity and increase the bleeding risk of anticoagulant therapy, some studies may have overestimated the safety risks associated with anticoagulation. Additionally, pulmonary hypertension, a common complication of IPF, independently increases mortality; failure to stratify for this condition may confound the true efficacy of anticoagulant therapy.

Thus, further analysis of the other five trials (excluding warfarin) showed that anticoagulants did reduce mortality. These findings suggest that the choice of anticoagulant may influence mortality in IPF. Variations in the type, dosage, and study design of anticoagulants may contribute to the heterogeneity observed in meta‐analytic outcomes. For example, heparin (5000 U) is administered subcutaneously, recombinant human soluble thrombomodulin is given intravenously at a dose of 0.06 mg/kg, while warfarin and direct oral anticoagulants (DOACs) are taken orally—a route that may improve patient adherence. Treatment durations also varied from 3 months to 1 year without a standardized regimen, which may lead short‐term studies to underestimate long‐term survival benefits, thereby affecting the reliability of the findings. Anticoagulants primarily inhibit thrombin production and activity, thus reducing lung tissue damage [27]. In IPF, chronic inflammation is a key part of the disease process, where inflammatory cells infiltrate the lungs, causing repeated injury and repair cycles that contribute to fibrosis [28]. Warfarin works by inhibiting vitamin K metabolism, reducing clotting factors [29]. However, individual differences in metabolism and sensitivity to warfarin may lead to adverse reactions, increasing mortality risk in some patients [30]. Other anticoagulants, such as heparin, recombinant human soluble thrombomodulin, and DOACs, not only inhibit thrombin but also reduce the release of inflammatory mediators and prevent inflammatory cell accumulation. This may explain why these anticoagulants are more effective in reducing mortality compared to warfarin [31].

The potential mechanism by which anticoagulants influence mortality in IPF may be linked to an increased risk of bleeding, which can worsen the progression of fibrosis. A meta‐analysis of four studies reporting bleeding events confirmed a higher incidence of bleeding in the anticoagulant group. This risk may be associated with factors like age, gender, hypertension, heart disease, diabetes, pulmonary hypertension, pulmonary embolism, and deep vein thrombosis [32–34]. Anticoagulants prevent blood clotting by inhibiting the production or activity of clotting factors. Given that IPF patients already have abnormal coagulation and endothelial damage, the integrity and stability of blood vessels may be compromised, increasing the risk of bleeding when anticoagulants are used.

Treatment outcomes in IPF are often assessed using markers like FVC, DLCO, and 6MWT. Our study found that anticoagulants improved FVC but had no significant impact on DLCO or 6MWT. These findings differ from previous studies [35, 36]. The pronounced heterogeneity in FVC outcomes may be closely related to the extent of lung impairment in patients. Individuals with mild to moderate impairment may benefit from anticoagulant therapy through improved pulmonary microcirculation and slower fibrosis progression. In contrast, patients with severe impairment often exhibit irreversible structural remodeling of lung tissue, making it difficult for anticoagulants to reverse the pathological process. This disparity likely contributes further to the observed heterogeneity [37]. DLCO reflects the lung’s gas exchange capacity and is closely related to factors such as the surface area and thickness of the alveolar membrane and blood circulation. In advanced stages of the disease, irreversible changes in lung tissue may prevent the reversal of these pathological alterations [38]. In addition to impaired lung function, 6MWT performance may also be influenced by factors such as muscle weakness, fatigue, and age, which can contribute to reduced exercise tolerance [39] that is possibly due to variations in study design or treatment interventions. Thus, multiple indicators should be considered in lung function assessments, along with clinical symptoms, for accurate diagnosis.

This meta‐analysis has several strengths, including a comprehensive search of the database and rigorous data extraction and assessment. The study highlights the impact of anticoagulants on survival rates and bleeding risks in IPF patients, providing new insights for future clinical or animal studies. It also lays a solid foundation for the combined use of anticoagulants and antifibrotic drugs in IPF treatment.

However, this study has some limitations. First, there was no standardized use of anticoagulants across the studies, and the heterogeneity between trials prevented meta‐analysis of certain outcomes. Second, subgroup analyses of different anticoagulant types should have been conducted, but the limited number of studies and lack of comparative trial data made this difficult.

Despite these limitations, we believe this meta‐analysis makes a valuable contribution to the literature on anticoagulant use in IPF treatment. Future high‐quality controlled trials are needed to identify the optimal anticoagulant for IPF patients, and our findings offer a new direction for subsequent research.

## 5. Conclusion

This standardized, high‐quality randomized controlled trial employed a unified protocol to more accurately evaluate the safety and efficacy of different anticoagulants in patients with IPF. The results indicate that the use of nonwarfarin anticoagulants may prolong survival in patients with IPF and potentially improve lung function. Compared with the control group, adverse events associated with anticoagulant therapy may be attributable to either the underlying disease or the drug’s side effects. In summary, selecting anticoagulants with a more favorable safety profile may guide future research directions and inform clinical decision‐making.

**Nomenclature**
IPF: Idiopathic pulmonary fibrosis
FVC: Forced vital capacity
6WNT: 6‐min walk test
DLCO: Diffusing capacity for carbon monoxide
BLM: Bleomycin
OR: Odds ratios
CI: Confidence intervals

## Ethics Statement

All summary statistics used in this MR analysis were obtained from public databases and published studies. The original studies received ethical approval and obtained individual consent.

## Consent

The authors have nothing to report.

## Disclosure

All authors read and approved the final manuscript.

## Conflicts of Interest

The authors declare no conflicts of interest.

## Author Contributions

S.Y., X.X., and D.X. designed the study and drafted the manuscript. Z.Y. and J.W. performed the data collection and analysis.

## Funding

This study was supported by the National Natural Science Foundation of China (Item number, 82374414) and Shanxi Provincial Department of Science and Technology (Item number, 202303021211239).

## Supporting information


**Supporting Information 1** 1. Search queries.

**Supporting Information 2** 2. PRISMA checklist.

## Data Availability

The authors have nothing to report.
