# Supplementary material for: Efficacy and Safety of Anticoagulants in Patients With Idiopathic Pulmonary Fibrosis: A Meta‐Analysis
Source: Can Respir J. 2025 Dec 14;2025:4545181. doi: 10.1155/carj/4545181 (PMC12714097; doi:10.1155/carj/4545181)
Supplement: Supplementary file 1 — Supporting Information 1 1. Search queries. [file CARJ-2025-4545181-s001.docx]

**Search queries**

| **Pubmed** |
| --- |
| ( (Anticoagulant Drug) OR ( Drug, Anticoagulant) OR ( Anticoagulant Agent) OR ( Agent, Anticoagulant) OR ( Anticoagulation Agents) OR ( Agents, Anticoagulation) OR ( Anticoagulant Drugs) OR ( Drugs, Anticoagulant) OR ( Anticoagulant Agents) OR ( Agents, Anticoagulant) OR ( Anticoagulant) OR ( Indirect Thrombin Inhibitors) OR ( Inhibitors, Indirect Thrombin) OR ( Thrombin Inhibitors, Indirect)) AND ((Idiopathic Pulmonary Fibroses) OR ( Pulmonary Fibroses, Idiopathic) OR ( Cryptogenic Fibrosing Alveolitis) OR ( Cryptogenic Fibrosing Alveolitides) OR ( Fibrosing Alveolitides, Cryptogenic) OR ( Pulmonary Fibrosis, Idiopathic) OR ( Fibrosing Alveolitis, Cryptogenic) OR ( Fibrocystic Pulmonary Dysplasia) OR ( Dysplasia, Fibrocystic Pulmonary) OR ( Fibrocystic Pulmonary Dysplasias) OR ( Pulmonary Dysplasia, Fibrocystic) OR ( Idiopathic Fibrosing Alveolitis, Chronic Form) OR ( Familial Idiopathic Pulmonary Fibrosis) OR ( Idiopathic Pulmonary Fibrosis, Familial) OR ( Usual Interstitial Pneumonia) OR ( Interstitial Pneumonia, Usual) OR ( Usual Interstitial Pneumonias) OR ( Interstitial Pneumonitis, Usual) OR ( Pneumonitides, Usual Interstitial) OR ( Pneumonitis, Usual Interstitial) OR ( Usual Interstitial Pneumonitides) OR ( Usual Interstitial Pneumonitis) ) |

| **Web of Science** |
| --- |
| ( (Anticoagulant Drug) OR ( Drug, Anticoagulant) OR ( Anticoagulant Agent) OR ( Agent, Anticoagulant) OR ( Anticoagulation Agents) OR ( Agents, Anticoagulation) OR ( Anticoagulant Drugs) OR ( Drugs, Anticoagulant) OR ( Anticoagulant Agents) OR ( Agents, Anticoagulant) OR ( Anticoagulant) OR ( Indirect Thrombin Inhibitors) OR ( Inhibitors, Indirect Thrombin) OR ( Thrombin Inhibitors, Indirect)) AND ((Idiopathic Pulmonary Fibroses) OR ( Pulmonary Fibroses, Idiopathic) OR ( Cryptogenic Fibrosing Alveolitis) OR ( Cryptogenic Fibrosing Alveolitides) OR ( Fibrosing Alveolitides, Cryptogenic) OR ( Pulmonary Fibrosis, Idiopathic) OR ( Fibrosing Alveolitis, Cryptogenic) OR ( Fibrocystic Pulmonary Dysplasia) OR ( Dysplasia, Fibrocystic Pulmonary) OR ( Fibrocystic Pulmonary Dysplasias) OR ( Pulmonary Dysplasia, Fibrocystic) OR ( Idiopathic Fibrosing Alveolitis, Chronic Form) OR ( Familial Idiopathic Pulmonary Fibrosis) OR ( Idiopathic Pulmonary Fibrosis, Familial) OR ( Usual Interstitial Pneumonia) OR ( Interstitial Pneumonia, Usual) OR ( Usual Interstitial Pneumonias) OR ( Interstitial Pneumonitis, Usual) OR ( Pneumonitides, Usual Interstitial) OR ( Pneumonitis, Usual Interstitial) OR ( Usual Interstitial Pneumonitides) OR ( Usual Interstitial Pneumonitis) ) |

| **Embase** | |
| --- | --- |
| **1** | 'Idiopathic Pulmonary Fibroses' OR ' Pulmonary Fibroses, Idiopathic' OR ' Cryptogenic Fibrosing Alveolitis' OR ' Cryptogenic Fibrosing Alveolitides' OR ' Fibrosing Alveolitides, Cryptogenic' OR ' Pulmonary Fibrosis, Idiopathic' OR ' Fibrosing Alveolitis, Cryptogenic' OR ' Fibrocystic Pulmonary Dysplasia' OR ' Dysplasia, Fibrocystic Pulmonary' OR ' Fibrocystic Pulmonary Dysplasias' OR ' Pulmonary Dysplasia, Fibrocystic' OR ' Idiopathic Fibrosing Alveolitis, Chronic Form' OR ' Familial Idiopathic Pulmonary Fibrosis' OR ' Idiopathic Pulmonary Fibrosis, Familial' OR ' Usual Interstitial Pneumonia' OR ' Interstitial Pneumonia, Usual' OR ' Usual Interstitial Pneumonias' OR ' Interstitial Pneumonitis, Usual' OR ' Pneumonitides, Usual Interstitial' OR ' Pneumonitis, Usual Interstitial' OR ' Usual Interstitial Pneumonitides' OR ' Usual Interstitial Pneumonitis' |
| **2** | 'Anticoagulant Drug' OR ' Drug, Anticoagulant' OR ' Anticoagulant Agent' OR ' Agent, Anticoagulant' OR ' Anticoagulation Agents' OR ' Agents, Anticoagulation' OR ' Anticoagulant Drugs' OR ' Drugs, Anticoagulant' OR ' Anticoagulant Agents' OR ' Agents, Anticoagulant' OR ' Anticoagulant' OR ' Indirect Thrombin Inhibitors' OR ' Inhibitors, Indirect Thrombin' OR ' Thrombin Inhibitors, Indirect' |
| **3** | 1 AND 2 |

| **Cochrane** | |
| --- | --- |
| **#1** | [Anticoagulants] explode all trees |
| **#2** | [Idiopathic Pulmonary Fibrosis] explode all trees |
| **#3** | #1 AND #2 |
